# Use of logit transformation within statistical analyses of experimental results obtained as proportions: example of method validation experiments and EQA in flow cytometry

**DOI:** 10.3389/fmolb.2024.1335174

**Published:** 2024-07-11

**Authors:** S. Seiffert, S. Weber, U. Sack, T. Keller

**Affiliations:** ^1^ Medical Faculty, Institute of Clinical Immunology, University of Leipzig, Leipzig, Germany; ^2^ ACOMED statistik, Leipzig, Germany

**Keywords:** external quality assessments (EQA), logit transformation, flow cytometry, method validation, proportions

## Abstract

In laboratory medicine, measurement results are often expressed as proportions of concentrations or counts. These proportions have distinct mathematical properties that can lead to unexpected results when conventional parametric statistical methods are naively applied without due consideration in the analysis of method validation experiments, quality assessments, or clinical studies. In particular, data points near 0% or 100% can lead to misleading analytical conclusions. To avoid these problems, the logit transformation—defined as the natural logarithm of the proportion/(1-proportion)—is used. This transformation produces symmetric distributions centered at zero that extend infinitely in both directions without upper or lower bounds. As a result, parametric statistical methods can be used without introducing bias. Furthermore, homogeneity of variances (HoV) is given. The benefits of this technique are illustrated by two applications: (i) flow cytometry measurement results expressed as proportions and (ii) probabilities derived from multivariable models. In the first case, naive analyses within external quality assessment (EQA) evaluations that lead to inconsistent results are effectively corrected. Second, the transformation eliminates bias and variance heterogeneity, allowing for more effective precision estimation. In summary, the logit transformation ensures unbiased results in statistical analyses. Given the resulting homogeneity of variances, common parametric statistical methods can be implemented, potentially increasing the efficiency of the analysis.

## Introduction

Achieving harmonization and improving the quality of measurements are central goals within the laboratory medicine community. Often, there are a number of measurement methods available for a given measurand from different manufacturers and laboratories. In addition, for methods such as flow cytometry, different experimental settings are used to assess the same measurand. This includes the use of different antibodies and gating strategies that vary from laboratory to laboratory.

In view of this situation, method developers and clinical laboratories are highly motivated to evaluate their measurement methods in internal method validation to gain knowledge about systematic and random errors [Lambert et al., 2020, CLSI guidelines, e.g., EP05]. In addition, external quality assessments (EQAs) are routinely performed.

Method validation typically includes method comparison and precision assessment. It is also important to demonstrate detection capability, robustness to interferences, etc. These experiments are typically analyzed using parametric statistical methods that rely on estimates of the mean and standard deviation (SD) for calculations. However, these methods assume that the data distribution should not deviate significantly from the normal distribution (ND) and that homogeneity of variances (HoV) is maintained over the measurement range, implying that precision is not concentration-dependent. In terms of ND, a visual inspection would be expected to show a symmetrical, bell-shaped distribution with no outliers. In this context, ISO 13528, which guides the design and analysis of EQA, specifies the need for symmetrical distributions.

In the context of EQA, a distinction is made between methods that use reference material and those that do not. If no reference material is available, samples supplied by a service provider are measured by all participating laboratories. The distribution of measurement results is considered, and an *assigned value*—calculated as a robust mean (ISO, 2022; Appendix C, Algorithm A)—is derived directly from the measurements of the participating laboratories. Such assigned values are then used as the basis for individual pass/fail assessments. The establishment of specific pass/fail criteria often involves a balance between observed measurement variability, quality requirements, and the clinical relevance of potential differences. We focus on the common scenario where acceptance criteria are defined by a relative or percentage difference around the *assigned value*.

For example, a typical EQA scenario might assess whether individual laboratories pass if their measurement values for a particular sample are within ± 30 percent of the robust mean (ISO, 2022; Section. 9.3).

The assumptions regarding ND (symmetry) and HoV should be met in such assessments. Although typical laboratory measurands measured in concentrations usually satisfy these assumptions, measurands related to inflammation or tumor incidence are often skewed to the right. In such cases, logarithmic transformation (or, more generally, Box–Cox transformation of the measured values) can help achieve a distribution that does not deviate significantly from the normal distribution and maintains homogeneity of variances. For count data, such as cell counts, square root transformation is often beneficial.

In this context, we consider measurands measured as proportions (0%–100%) or probabilities (0–1) that represent relative measures of specific subgroups within an entity. In flow cytometry, such measurands are exemplified by the evaluation of CD3^+^ cell subsets, specifically alpha/beta T cells and gamma/delta T cells, both quantified as a percentage of CD3^+^ cells. Since their combined values add up to 100%, the measured values are inherently correlated.

Another example is the use of probabilities as metrics, such as calculated measurands resulting from multivariable analysis of measured values from a variety of measurands. These probabilities may be generated by logistic regression or other classification methods, such as machine learning or artificial intelligence.

In both scenarios, EQA and combined markers, proportions, and probabilities are bounded between 0% and 100%. Note that in this article, we also use 0 and 1 (representing percentages divided by 100), depending on the context. Because the data are constrained by 0% and 100%, the assumptions regarding ND and HoV are no longer valid, especially for values approaching the limits (<20% and >80%). Consequently, describing, analyzing, or statistically testing experimental data using parametric methods—such as calculating mean and standard deviation, performing *t*-tests and ANOVA for group differences, and estimating variance components (precision) or using ordinary regression techniques—will yield invalid results. This is because these methods assume ND and HoV. In addition, the use of symmetric power limits becomes untenable.

Nevertheless, the naive application of parametric methods to proportions or probabilities is often observed, often due to the convenience of ready-to-use software packages in daily routine and the seemingly straightforward interpretation of results.

Although nonparametric methods could be an alternative, they are less powerful, often require larger sample sizes, and may not provide well-known estimates of bias and precision.

Therefore, we propose to logit transform measured values prior to statistical analysis with parametric methods when measurands are measured as proportions (or probabilities). This article highlights the differences between naive analysis and analysis using logits and provides guidance for interpreting the results.

## Materials and methods

### EQA data

Simulated data (26 laboratories) from an EQA for alpha/beta T cells (% of CD3^+^) with an assumed true measured value of 95% and gamma/delta T cells (% of CD3^+^) with an assumed true measured value of 5% are used. The simulation generates a random number for each laboratory by adding normally distributed noise with an SD of 0.2944, distributed around the assumed value on the logit scale (95% → 2.944). The simulated distribution reflects typical scenarios encountered in the INSTAND program for flow cytometry. In our considerations, we assume that both measurands are highly correlated. For the sake of simplicity, we have assumed a direct relationship between the variables, ensuring that the percentages add up to 100%. Thus, the negative values of measurand A are used as values for measurand B. We have assumed the absence of outliers in this simulation, as outlier detection is beyond the scope of this report.

### Precision data

In this simulation study, we generated data representing the results obtained as probabilities from an automated biomarker measurement procedure. Specifically, we measured these probabilities on five different days, with triplicate measurements performed on six different samples. The aim of the experiment is to determine repeatability and overall precision, following the methodology outlined in CLSI (2014).

However, due to the small sample size (15 measurements instead of the recommended 80 measurements for one sample), we chose to estimate repeatability and overall precision pooled over the samples, as suggested by Lambert et al. (2022). This pooling approach assumes the homogeneity of variances.

Within the simulation, the samples were assigned the following values: 4.7%, 14.2%, 37.4%, 64.6%, 85.8%, and 95.3%. The simulation was designed to start with homogeneous variances on a logit scale and then illustrate how the data would be represented on the original probability scale. Within the simulation, the components of imprecision are addressed by adding noise using normal distributed random numbers with the following standard deviations. Within the logit scale, the repeatability (the variability within 1 day) was set at 0.20 for all samples, expressed as the standard deviation. At the same time, the between-day variability was set at 0.12 for all samples. The resulting reproducibility is 0.233 (the square root of the sum of the squared SDs).

To illustrate the impact of using probabilities versus logits, we present variability plots and analyze the precision experiment both naively (per sample) and using logit-transformed values pooled across samples.

The precision components were estimated using random effects ANOVA, as described in CLSI (2014).

## Results

### Logit transformation

In the logit transformation of a proportion (or probability) p, the following formula is used:

$$\text{logit}\left(p\right)=\ln\left(\frac{p}{1-p}\right),$$ (1)

where ln is the natural logarithm.

Table 1 contains selected probabilities and their corresponding logit values that are often encountered in daily work.

**TABLE 1:** Examples for logit transformation.

p	Percentage (%)	Logit(p)
0.01	1	−4.60
0.05	5	−2.94
0.1	10	−2.20
0.5	50	0
0.9	90	2.20
0.95	95	2.94
0.99	99	4.6

For the back transformation, the following formula is used:

$$p=\text{antilogit}\left(x\right)=\frac{\exp\left(x\right)}{1+\exp\left(x\right)},$$ (2)

where x is a value on the logit scale.

Supplementary Material includes an Excel tool for these calculations (Supplementary Material S2).

### Application in the context of EQA

In the EQA experiments described above, naive analysis typically involves (i) the calculation of the mean and (ii) the symmetric setting of limits, which is flawed when the underlying distribution is asymmetric. The bias is evident in the calculation of limits, such as the mean ± 30% of the mean, for related percentages, such as 5% and 95%. In a naive analysis, the bounds would be 3.5% … 6.5% for the 5% mean and 66.5% … 100% for the 95% mean.

To calculate accurate limits using the logit scale, the following formula (in conjunction with Eqs 1, 2) is used:

$$\pm {Limit}_{Logit-based}=antilogit\left(logit\left(p\right)\pm logit\left({Limit}_{Orig}\right)\right).$$ (3)

**FIGURE 1:**
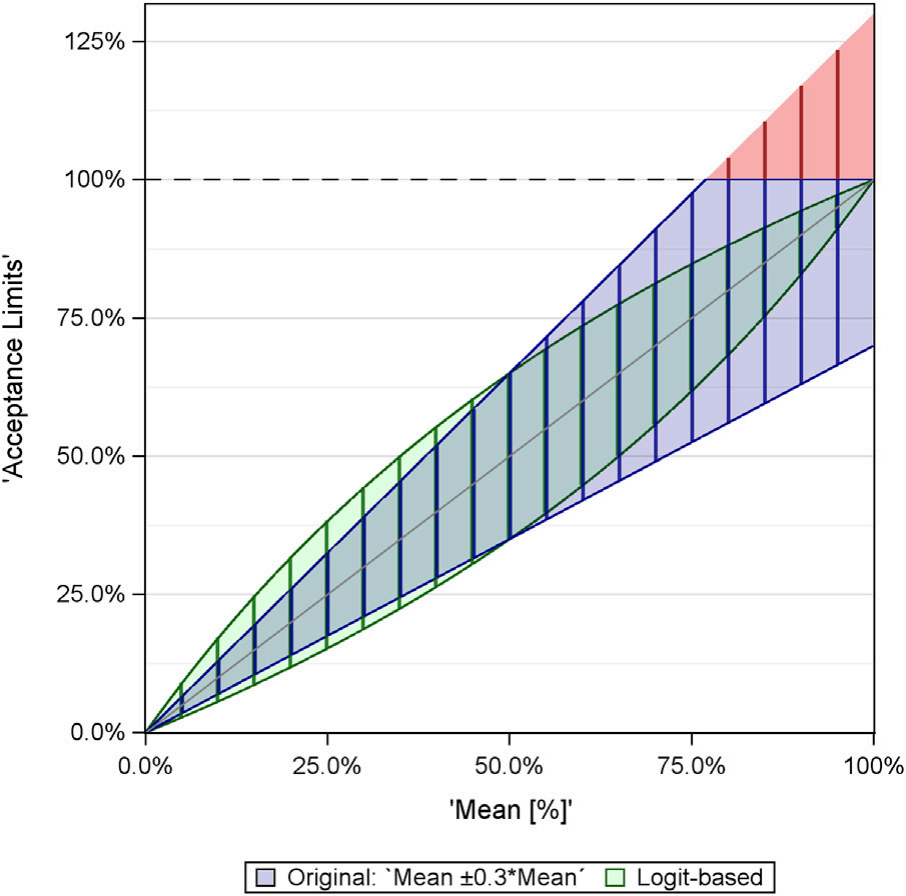
Comparison of limits derived within naive EQA analysis (blue/red) and analysis via logit-transformed values (green). The red color refers to the limits >100%, which are not meaningful and are set to 100%.

Figure 1 illustrates the difference between the limits derived from the naive analysis and those obtained using the logit transformation, using the example mean ± 30% x mean. For the 5% and 95% percentages, the ranges between the limits become identical: 3.5% … 6.5% for 5% and 93.5% … 96.5% for 95%.

Supplementary Material S3 contains an Excel tool for deriving limits on the logit scale.

**FIGURE 2:**
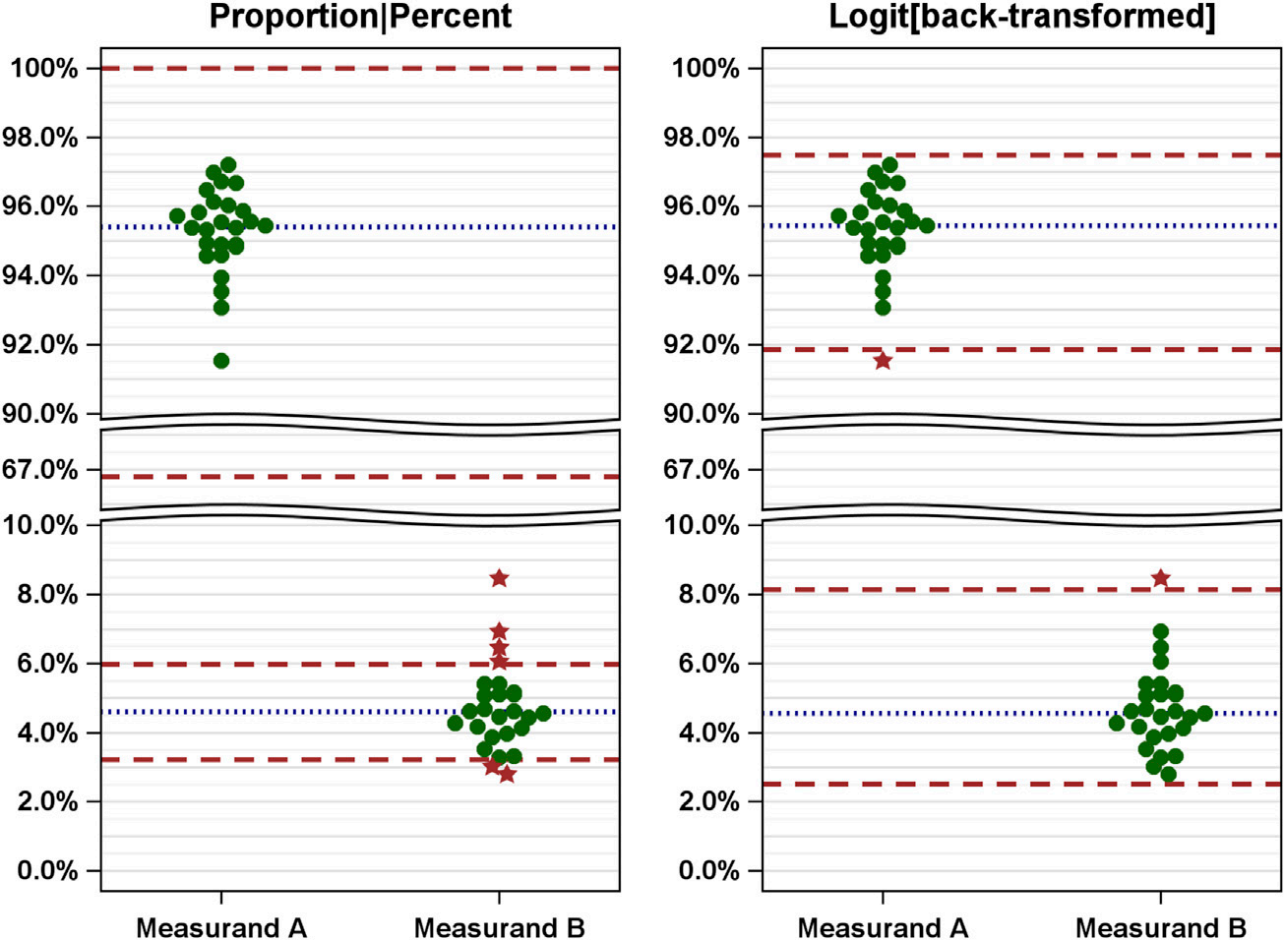
Comparison of EQAs (measured values and limits) based on naive analysis (left) and improved analysis (right) using simulated measured results of two measurands [A, alpha/beta T cells (% of CD3^+^); B, gamma/delta T cells (% of CD3^+^)]. The measured values are shown as dots and related to the naively (left: proportions) and correctly (right: logit-scaled values) calculated limits. Green dots, passed; red stars, failed.

The following example illustrates the consequences of EQAs. Figure 2 shows simulated data against limits derived naively (left) and after logit transformation of the data and back transformation of the means and limits (right). Supplementary Table S1 lists all values and the EQA results.

The simulation yields skewed distributions with mean values of 95.4% for measurand A and 4.6% for measurand B. In a naive analysis, the limits are between 66.8% and 100% for measurand A and 3.2% and 6.0% for measurand B (Figure 2, left). The span of the ranges defined by these limits varies considerably. Using these limits, all results for measurand A were considered valid, while six results for measurand B were considered invalid.

Conversely, when logit-transformed values are used, the means are nearly identical (see Supplementary Table S1 for details). The limits (calculated according to Eq. 3) are 91.8%–97.5% for measurand A and 2.5%–8.2% for measurand B. Notably, these limits are not symmetric with respect to the mean, but the width of the range defined by the limits is the same (5.7%), as shown in Figure 2 (right). Using these limits, one value for each measurand was identified as invalid.

### Application for precision evaluation

In a simulated precision experiment conducted over 5 days, 6 samples were measured in triplicate.

**FIGURE 3:**
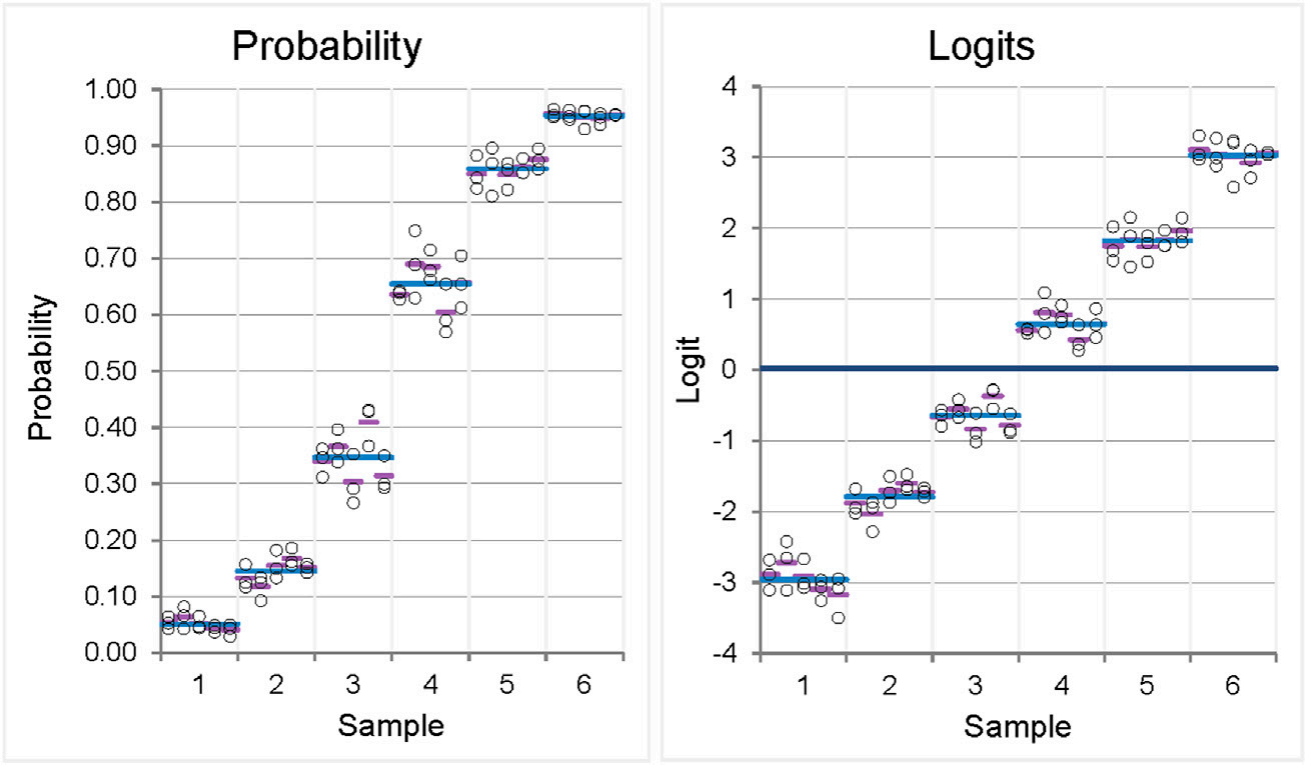
Variability plots for originally scaled (left) and logit-scaled (right) measured values of a precision experiment showing homogeneity of variances (homoscedasticity) when logit-transformed values are used for analysis, whereas inhomogeneity of variances is observed when originally scaled proportions are used. The measured values result from a simulation of a 6-sample, ×5-day, ×3-replicates’ precision experiment with given values for repeatability and reproducibility on the logit scale (0.20, 0.12). Each circle represents one measurement; pink, mean of the replicates within 1 day; blue, mean of the sample.

As mentioned earlier, the simulation starts with a given level of repeatability and between-day precision on the logit scale. The associated standard deviations (SDs) in the simulation results show little variation due to random error, as shown in Figure 3 (left). The standard deviations derived from the naive analysis on the probability scale, which represents the scale on which the values would be measured, vary (Figure 3, right). Heterogeneity of the standard deviations is observed, ranging from 0.010 to 0.041 (Table 2, left column; see below for further explanation).

**TABLE 2:** Results of precision analysis [repeatability and reproducibility expressed as the standard deviation (SD)/95% confidence intervals (CIs)] calculated by random effects ANOVA as described in CLSI EP05A3. Left: naive analysis per sample on the probability scale; right: analysis pooled across samples on the logit scale, which is possible if homogeneity of variances across samples is given. The results of the pooled analysis are presented on the logit scale and back-transformed to the probability scale for each sample. Since the distribution of the measured values is not symmetric, except for a probability of 0.5, the lower and upper SDs are presented. For a probability of 0.5, only one SD is reported.

Sample	Analysis based on								
	Probability (naïve analysis) analysis per sample			Logit values, pooled analysis					
				Pooled (on the logit scale)		Pooled analysis back-transformed on each sample level			
	Mean	Repeatability 95% CI	Reproducibility 95% CI	Repeatability 95% CI	Reproducibility 95% CI	Repeatability		Reproducibility	
						Lower SD 95% CI	Upper SD 95% CI	Lower SD 95% CI	Upper SD 95% CI
n.a				0.2042 0.1733–0.2486	0.2232 0.1956–0.2728				
1	0.049	0.012 0.009–0.022	0.013 0.010–0.028	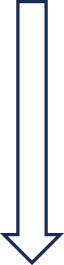		0.009 0.007–0.010	0.010 0.009–0.013	0.009 0.008–0.011	0.012 0.010–0.014
2	0.143	0.019 0.013–0.034	0.025 0.019–0.060			0.023 0.020–0.028	0.027 0.023–0.033	0.025 0.022–0.030	0.030 0.026–0.037
3	0.345	0.034 0.024–0.060	0.051 0.037–0.125			0.045 0.038–0.054	0.047 0.040–0.058	0.049 0.043–0.059	0.052 0.045–0.064
*n.a*	*0.5*			*The pooled results can be back-transformed* and *reported as the mean* ± *SD for a probability of 0.5*		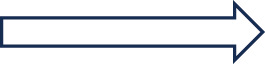 0.051 0.043–0.062		0.056 0.049–0.068	
4	0.656	0.041 0.029–0.072	0.049 0.037–0.108			0.047 0.040–0.058	0.045 0.038–0.054	0.052 0.045–0.064	0.048 0.043–0.059
5	0.861	0.028 0.020–0.049	0.028 0.023–0.046			0.026 0.022–0.033	0.023 0.019–0.027	0.029 0.025–0.036	0.025 0.022–0.030
6	0.954	0.010 0.007–0.018	0.010 0.008–0.017			0.010 0.008–0.012	0.008 0.007–0.010	0.011 0.009–0.014	0.009 0.008–0.011

It is important to note that the pooled standard deviations ${SD}_{pooled}$ derived from logit-transformed values require back transformation. The back-transformed standard deviation based on the logit transformation is now expressed as the lower and upper bounds of the standard deviation. The calculation is performed using the following formula:

$$p\pm {SD}_{logit-based}=antilogit\left(logit\left(p\right)\pm {SD}_{pooled}\right).$$ (4)

From Eq. 4, it follows that there are asymmetric “standard uncertainty ranges” [of 1 x SD width] obtained for “above” (see Eq. 5a) and “below” (see Eq. 5b) nominal p ranges.

$${SD}_{Logit-based}=antilogit\left(logit\left(p\right)+{SD}_{pooled}\right)-p,$$ (5a)

$${SD}_{Logit-based}=p-antilogit\left(logit\left(p\right)-{SD}_{pooled}\right).$$ (5b)

Appendix 4 provides an Excel spreadsheet that can be used for these calculations.

Table 2 shows these details of the precision analysis, comparing the naive analysis based on probabilities (left) with the analysis based on logit-scaled scores. In addition, the table shows the back-transformed results. Comparing the SDs from the naive and logit-based analyses at the probability level, it can be seen that the naive analysis leads to biased results.

Most important is the pooled precision across all samples, which is highlighted in the gray cells. This pooled analysis across samples is possible due to the homogeneity of the SDs on the logit scale (as shown in Figure 3, left).

The results of the pooled analysis can be presented in three ways: first, the pooled repeatability and reproducibility are expressed as standard deviations on a logit scale. Second, the back-transformed mean−SD and mean + SD at 50% probability are used. Since the mean−SD and mean + SD are equidistant from 50%, the standard deviation can be presented as a single value. This result can be used as an overall measure of the precision of the measurement procedure. Third, the back transformation of the mean−SD and mean + SD is performed at each sample level; here, the lower and upper SD are presented since the distribution of the measured values is not symmetric.

The results include 95% confidence intervals (CIs), which indicate the uncertainty of the repeatability and reproducibility estimates. The CIs for the pooled analysis are significantly narrower.

**FIGURE 4:**
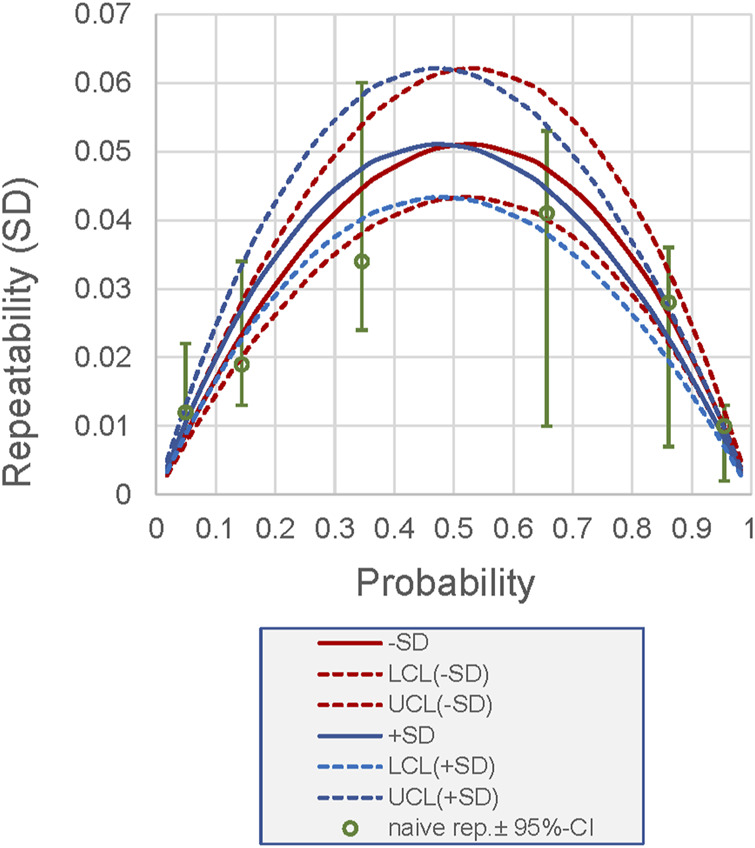
Relationship between probability (as a measurement result of a measurand measured as a proportion or probability) and its repeatability for the example shown in Figure 3. The repeatability is derived from the pooled analysis using logit-scaled values. Back-transformed values are presented. Lines, back-transformed SDs (+, −) derived from pooled repeatability; dashed lines, 95% CI. Dots, sample-specific repeatability derived from naive analysis (which does not allow pooled estimation), including 95% CI. LCL, lower confidence limit; UCL, upper confidence limit.

The back transformation of the pooled values of repeatability and reproducibility to the logit scale, as performed above for the 50% probability, is possible for any probability. Figure 4 illustrates this calculation. The curves allow the precision for each probability to be determined based on the pooled precision. In Figure 4, the results are compared with the results of the naive analysis, highlighting the discrepancies in the SDs and the wider CIs of the naive analysis.

## Discussion

We report on the use of the logit transformation to handle measured values given as proportions or probabilities before applying statistical analyses.

Our results indicate that naive analyses can lead to biased results, especially when the probabilities are close to the limits of 0 (0%) or 1 (100%). In these sub-ranges, the distributions of the values exhibit skewness, as shown in Figure 2 (left). To address these limitations, we suggest using the logit transformation. Analyses are then performed on the logit-transformed probabilities or proportions.

It is important to note that logit transformation is not a novel approach. The literature on predictive models often recommends the use of logit values instead of probabilities (Steyerberg, 2019), such as for model calibration (Ojeda et al., 2023). Furthermore, logit transformation is a special case for transforming data in beta regression, a suggested method for analyzing data observed in (0, 1) intervals (Kischinck and McCullough, 2003; Geissinger et al., 2022).

The use of the logit transformations, among other possible transformations (arcsin (square root), probit, and Fisher’s transformation), is also justified by its application in the statistical modeling of binary test results (numbers 0 and 1). One of the most effective methods for this purpose is logistic regression. As internal continuous outcomes, one obtains values on the logit scale, which are then transformed into probabilities to make the results more interpretable. In this context, the application of the logit transformation is straightforward; it is simply the inverse of the transformation. Calculated parameters expressed as probabilities could just result from this or similar classification procedures, so their relationship to logit values, the values calculated in the logistic regression model function, is quite obvious.

It should be noted that differences based on logits can be interpreted as odds ratios, but this is not of interest in the context of this paper, which focuses on the internal and external validation of a measurement method. However, when used in clinical research (e.g., clinical outcome studies), logit transformation provides additional and familiar ways of reporting results.

The use of logit-transformed probabilities is even mentioned in statistical textbooks for medical research (Bland, 2015; Armitage et al., 2015). However, we found no examples of its use in the laboratory medicine literature. This may be because the benefits of the logit transformation are fully realized when the entire range of probabilities, both below and above 50%, as well as near the 0% and 100% limits, is utilized by the measurand. Single measurands typically do not cover this range. However, the two examples in this publication illustrate this scenario. In the case of EQA, the results of the analysis of two corresponding measurands with readings close to 0% for the first and close to 100% for the second become consistent when the simple analysis is replaced by an analysis using the logit-transformed measured values. Another example of measurands that cover the entire measurement range is calculated parameters that reflect classification results based on multiple measurands (Keller et al., 1998; Klocker et al., 2020). Again, the advantage of the logit transformation is highlighted. In addition to providing unbiased estimates of precision, this scale ensures the homogeneity of variances, i.e., the random error does not systematically depend on the measured value. This allows the use of parametric statistical methods such as regression, ANOVA, or even pooling of precision across samples. The latter leads to a significant increase in statistical power or a reduction in the sample size required for the precision experiment.

Finally, we demonstrate the methodology on two examples from these areas: EQA of flow cytometry data measured as proportions and a method validation experiment (precision) on values expressed as probabilities. Figure 4 summarizes the advantages of logit-based precision analysis: it provides unbiased and more precise estimates of the precision components.

It is important to note that the analysis of other method validation experiments, such as method comparisons, can benefit significantly from our proposed approach.

Instead of using real data, we chose to use simulated data. Although this may be seen as a limitation of our study, it is important to note that these simulations were directly inspired by real examples from our daily work. In addition, we initially encountered naive analyses that had previously been used for statistical evaluations in the EQA program. Based on the considerations reported here, the EQA-related analyses are currently modified. In the case of the precision data of a calculated parameter, the precision is often found to be suboptimal and not meet the acceptance criteria. In that case, the variances of the contributing measurands add up due to error propagation. It is not the purpose of this article to report on these specific problematic data sets.

Moreover, real-world data often present their own challenges. In addition to the effect to be demonstrated, there may be other issues such as outliers, imperfect correlations, or dilution of effects due to simple imprecision. These complications require a discussion of all side effects, which can sometimes overshadow the main effects.

For these reasons, we decided to use simulated data.

When data transformations are applied, a thorough discussion of the back transformation is required. Although an unbiased analysis with the ability to use all the tools of parametric statistics is advantageous, it often comes at the cost of more complex, or at least unusual, handling of the results. For example, when standard deviations of proportions are evaluated, the results are more complex and require additional explanation. One option is to present the results on a logit scale, which may not be practical: this would require the user to be able to conceptualize in logits, which may not be a realistic expectation. However, when different values expressed as proportions are evaluated in parallel, it may be useful to compare the results directly on the logit scale.

Another option is to back-transform the results. It is important to note that only points (e.g., mean +SD and mean − SD) can be back-transformed, not a range such as SD. Due to the skewed distribution of the underlying values (proportions and probabilities), the resulting mean ± SD will also be asymmetric. Reporting the precision for a 50% probability as an abstract but easy-to-read overall measure is then advantageous because it is symmetric at approximately 50%.

Finally, we strongly recommend the use of logit-transformed data in statistical analyses of clinical laboratory and quality control data when the measures are proportions or probabilities. This approach enhances the interpretability and power of the results, thereby facilitating their application in the relevant fields.

## Data Availability

The original contributions presented in the study are included in the article/Supplementary Material; further inquiries can be directed to the corresponding author.
