# Supplementary material for: Use of logit transformation within statistical analyses of experimental results obtained as proportions: example of method validation experiments and EQA in flow cytometry
Source: Front Mol Biosci. 2024 Jul 11;11:1335174. doi: 10.3389/fmolb.2024.1335174 (PMC11269570; doi:10.3389/fmolb.2024.1335174)

SUPPLEMENTS

Supplement 1: Difference in EQA results (passed/failed) using proportion scale (left) and logit scale (right). For logit scale, the results for measurands A and B are consistent as one would expect. For original scale (proportions expressed in %), inconsistent results are yielded.
Simulated datasets for parameters X alpha/beta T-Cells [% of CD3+], assumed value of sample: 95% (measurand A) and gamma/delta T-Cells [% of CD3+], assumed value of the sample: 5% (measurand B). The footnote describes the derivation of the limits.

|  | **Measured values on % scale** | | | | **Measured values on Logit- scale** | | | |
| --- | --- | --- | --- | --- | --- | --- | --- | --- |
| **Laboratory** | **A** | | **B** | | **A** | | **B** | |
| 26 | 91.52% | passed | 8.477% | failed | 2.37926 | failed | -2.37926 | failed |
| 8 | 93.07% | passed | 6.933% | failed | 2.59705 | passed | -2.59705 | passed |
| 16 | 93.53% | passed | 6.470% | failed | 2.67114 | passed | -2.67114 | passed |
| 18 | 93.95% | passed | 6.055% | failed | 2.74183 | passed | -2.74183 | passed |
| 21 | 94.58% | passed | 5.421% | passed | 2.85920 | passed | -2.85920 | passed |
| 4 | 94.58% | passed | 5.421% | passed | 2.85921 | passed | -2.85921 | passed |
| 7 | 94.83% | passed | 5.170% | passed | 2.90928 | passed | -2.90928 | passed |
| 10 | 94.89% | passed | 5.110% | passed | 2.92142 | passed | -2.92142 | passed |
| 6 | 94.90% | passed | 5.105% | passed | 2.92255 | passed | -2.92255 | passed |
| 23 | 94.93% | passed | 5.067% | passed | 2.93034 | passed | -2.93034 | passed |
| 11 | 95.33% | passed | 4.674% | passed | 3.01534 | passed | -3.01534 | passed |
| 22 | 95.38% | passed | 4.622% | passed | 3.02691 | passed | -3.02691 | passed |
| 5 | 95.38% | passed | 4.620% | passed | 3.02750 | passed | -3.02750 | passed |
| 24 | 95.43% | passed | 4.568% | passed | 3.03939 | passed | -3.03939 | passed |
| 3 | 95.55% | passed | 4.454% | passed | 3.06585 | passed | -3.06585 | passed |
| 20 | 95.57% | passed | 4.434% | passed | 3.07043 | passed | -3.07043 | passed |
| 25 | 95.73% | passed | 4.271% | passed | 3.10973 | passed | -3.10973 | passed |
| 14 | 95.82% | passed | 4.176% | passed | 3.13322 | passed | -3.13322 | passed |
| 17 | 95.86% | passed | 4.142% | passed | 3.14157 | passed | -3.14157 | passed |
| 13 | 96.03% | passed | 3.969% | passed | 3.18615 | passed | -3.18615 | passed |
| 12 | 96.14% | passed | 3.864% | passed | 3.21407 | passed | -3.21407 | passed |
| 15 | 96.47% | passed | 3.528% | passed | 3.30860 | passed | -3.30860 | passed |
| 2 | 96.68% | passed | 3.322% | passed | 3.37078 | passed | -3.37078 | passed |
| 1 | 96.71% | passed | 3.292% | passed | 3.38032 | passed | -3.38032 | passed |
| 9 | 96.98% | passed | 3.017% | failed | 3.47037 | passed | -3.47037 | passed |
| 19 | 97.21% | passed | 2.791% | failed | 3.55063 | passed | -3.55063 | passed |
| **Acceptance criteria** **Measurand A** Proportion\|Percent:  95.4% [ 66.8% to   100%] Logit:   3.041 [  2.422 to   3.660] *Logit[back-transformed]:  95.4% [ 91.8% to  97.5%]* **Measurand B** Proportion\|Percent:  4.59% [ 3.22% to  5.97%] Logit:  -3.041 [ -3.660 to  -2.422] *Logit[back-transformed]:  4.56% [ 2.51% to  8.15%]* | | | | | | | | |

Supplement 2

Screenshot of an Excel sheet for logit transformation of proportions/probabilities and back-transformation


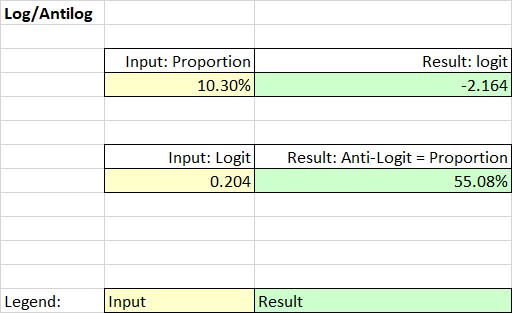


Supplement 3

Screenshot of an Excel sheet to derive limits in EQA


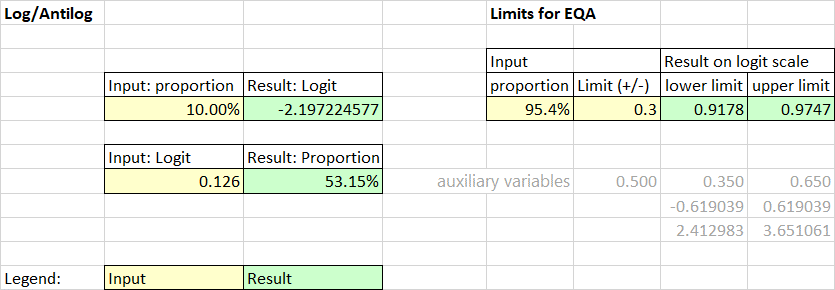


Supplement 4

Screenshot of an Excel sheet to calculate SD on probability scale based on analysis of logits.


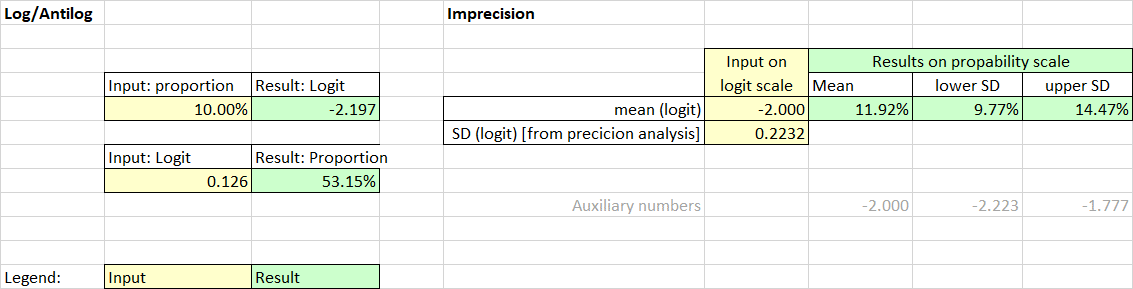

Supplement: Supplementary file 1 [file DataSheet1.docx]
